# Antibody-mediated rejection—treatment standard

**DOI:** 10.1093/ndt/gfaf097

**Published:** 2025-05-29

**Authors:** Georg A Böhmig, Maarten Naesens, Ondrej Viklicky, Olivier Thaunat, Matthias Diebold, Lionel Rostaing, Klemens Budde

**Affiliations:** Division of Nephrology and Dialysis, Department of Medicine III, Medical University of Vienna, Vienna, Austria; Department of Microbiology, Immunology and Transplantation, KU Leuven, Leuven, Belgium; Department of Nephrology, Institute for Clinical and Experimental Medicine, Prague, Czech Republic; Service de Néphrologie, Hémodialyse, Aphérèses et Transplantation, Centre Hospitalier Universitaire Grenoble-Alpes, France; Clinic for Transplantation Immunology and Nephrology, University Hospital Basel, University of Basel, Basel, Switzerland; Department of Transplantation, Nephrology and Clinical Immunology, Edouard Herriot Hospital, Hospices Civils de Lyon, Lyon, France; Department of Nephrology, Charité Universitätsmedizin Berlin, Berlin, Germany

**Keywords:** antibody-mediated rejection, apheresis, CD38, donor-specific antibody, natural killer cells

## Abstract

Antibody-mediated rejection (AMR) remains a major cause of graft failure, with significant health and economic burden. Despite being recognized >25 years ago, AMR treatment remains unstandardized, and no therapy has gained robust regulatory approval. While uncontrolled series have shown promise, few well-designed trials exist, with most yielding negative results. In the absence of strong trial data, a Transplantation Society expert consensus recommended potential treatment options with low levels of evidence, tailored to clinical phenotypes. Here, we re-evaluate the current evidence for AMR treatment decisions. We conclude that steroids, rituximab, bortezomib, and interleukin-6 (IL-6) antagonists lack sufficiently robust evidence to support their use in AMR. For early AMR, antibody depletion using immunoadsorption could be considered as an alternative to plasmapheresis. High-dose intravenous immunoglobulin (IVIG) may be added, though the supporting evidence remains limited. While previous trials primarily targeted the cause of AMR, recent data on the successful reversal of AMR activity by CD38 antibodies—particularly recent phase 2 trial results—suggest that targeting the cellular inflammation resulting from antibody binding to the endothelium could be a rational approach. Along these lines, in severe early AMR, complement inhibition may also be an option. Ongoing phase 2 trials evaluating prolonged courses of high-dose IVIG, the neonatal Fc receptor blocker efgartigimod, the tyrosine kinase inhibitor fostamatinib, and the complement inhibitor BIVV020, along with phase 3 trials of the anti-IL-6 receptor antibody tocilizumab and the CD38 antibody felzartamab, offer hope for effective, approved therapies targeting different aspects of AMR pathobiology.

In a nutshell:

- AMR remains a major cause of graft dysfunction and failure. Despite increasing insights into the biology of AMR, no robustly evidenced or regulatory-approved standard therapy currently exists. To date, several systematic trials have failed to demonstrate a convincing benefit for various therapeutic approaches targeting the pathophysiological mechanisms of AMR, including rituximab, bortezomib, and the anti-IL-6 antibody clazakizumab. Current recommendations for AMR treatment are based on expert opinion and limited evidence from case series, uncontrolled studies, and a small number of systematic trials.
- Building on the 2019 Expert Consensus from The Transplantation Society Working Group, we recommend apheresis—either plasmapheresis or immunoadsorption—as a mainstay in the treatment of early AMR, primarily caused by preformed donor-specific antibodies (DSA). Optionally, high-dose IVIG may be administered at the end of apheresis treatment. Additionally, we emphasize the optimization of immunosuppression as a key intervention, although it is unlikely to significantly affect antibody levels once the antibodies have already formed.
- Based on promising recent data, we propose considering strategies that target the downstream pathobiology of AMR, e.g. using CD38 antibody therapy to interfere with natural killer (NK) cell-triggered graft injury. Moreover, complement inhibition with eculizumab may be a potential option for severe early AMR.
- When considering intensified immunosuppressive measures, factors such as the activity and severity of rejection in relation to recipient age and comorbidities, as well as the level of irreversible chronic injury at biopsy—a key predictor of shortened graft survival—should guide treatment intensity.
- We recommend the inclusion of patients, when possible, in prospective interventional trials to systematically gather evidence for improved treatment of AMR. Several trials are currently underway, including phase 3 trials evaluating the CD38 antibody felzartamab and the anti-interleukin-6 receptor antibody tocilizumab that could counteract B cell alloimmunity and inflammation. Additionally, phase 2 trials are investigating prolonged courses of high-dose IVIG, the tyrosine kinase inhibitor fostamatinib and the FcRn blocker efgartigimod.

## INTRODUCTION

Kidney transplant rejection is classically categorized into two forms: T cell-mediated rejection (TCMR) and antibody-mediated rejection (AMR) [1, 2]. AMR, primarily caused by anti-HLA donor-specific antibodies (DSA), remains a leading cause of graft failure, with no approved treatment and limited management guidance [3]. According to the Banff classification, AMR is diagnosed based on DSA and/or capillary C4d detection, along with microvascular inflammation (MVI), in advanced cases, chronic injury in glomeruli or peritubular capillaries [4]. Morphologically, AMR presents as active, chronic-active, or chronic (inactive) subtypes [4]. Early AMR, often due to preformed DSA, may cause acute graft dysfunction, whereas *de novo* DSA-driven rejection may progress slowly over years. A systematic review of 28 studies reported AMR incidences between 3% and 12%, with chronic AMR affecting 7.5%–20.1% over 10 years [5]. Retrospective analyses identified AMR as a primary cause of graft failure, often linked to medication non-adherence [6, 7]. A US cohort study of 3131 recipients found 194 AMR patients faced a 10-fold higher hazard of death-censored graft failure despite treatment, with late AMR carrying even greater risk [8]. A study of 5679 patients with AMR highlighted the substantial socioeconomic and health impact of AMR, showing nearly triple the risk of graft failure and death at 2 years, with healthcare costs four times higher ($35 750 per patient annually in the 2 years after AMR diagnosis) [9].

This article explores therapeutic challenges and options in AMR based on current literature, and outlines our recommendations and suggestions within this context.

## TREATMENT STANDARDS

### General considerations

#### Lack of high-quality evidence

**Figure 1:**
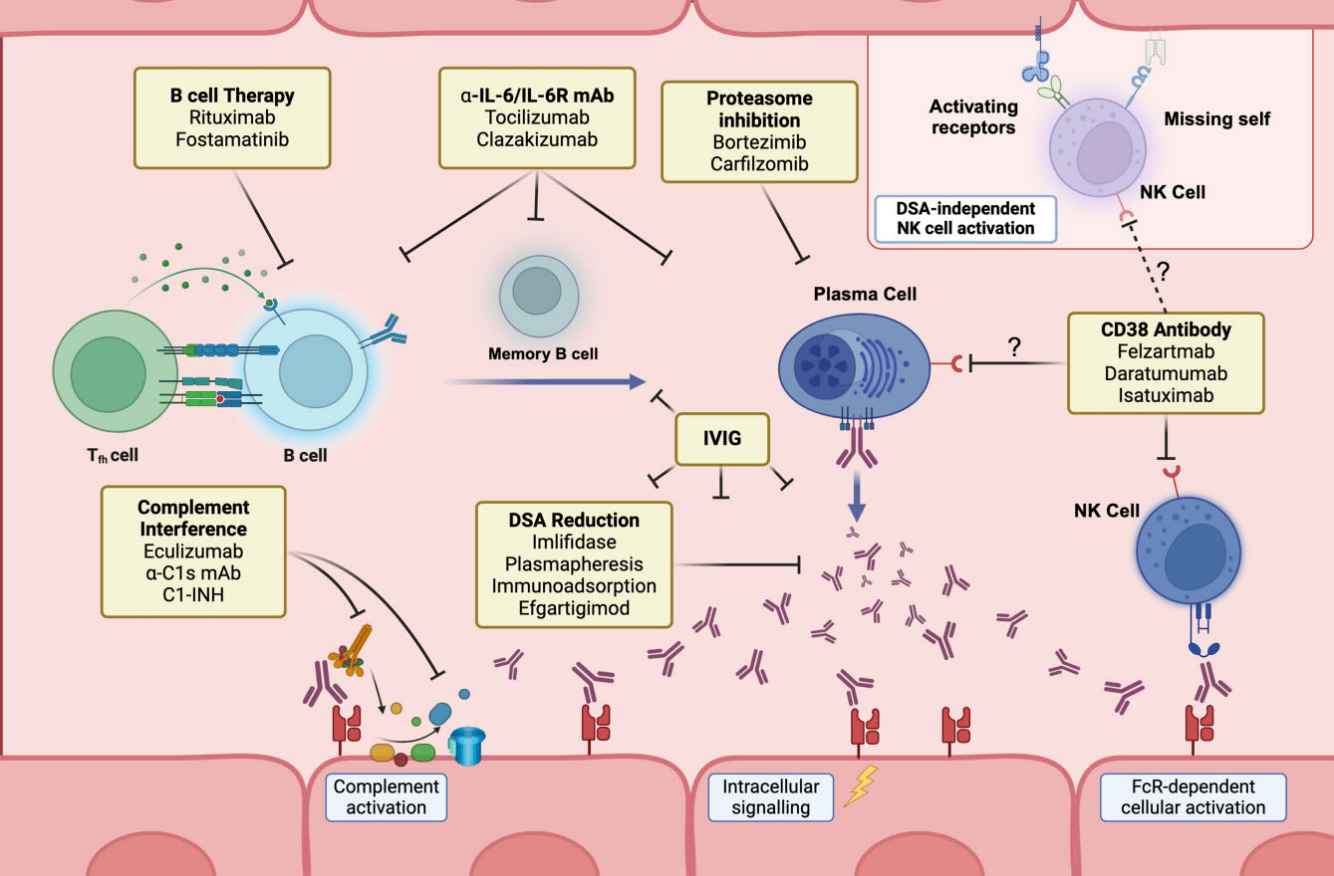
Pathophysiological sequence leading to AMR and DSA-negative MVI. Following B-cell activation and differentiation into antibody-producing plasma cells, DSA are produced. DSA bind to the endothelium of the microvasculature where they can lead to the histologic hallmark lesion of MVI, through complement activation, direct effects, or Fc gamma receptor-dependent effector cell activation. NK cells may be activated via Fc gamma receptor IIIA, or, alternatively, independently of DSA through missing self-recognition or distinct activating receptors. Potential therapeutic options targeting B cells and plasma cells, complement, DSA levels and/or NK cells are illustrated. Created in https://BioRender.com. Ab, antibody; IL-6R, interleukin-6 receptor, C1-INH, C1 esterase inhibitor.

The well-charted pathophysiologic sequence of AMR suggests multiple potential therapeutic targets and strategies (Fig. 1). However, no treatments are approved, and few therapies have progressed to efficacy trials, with none passing phase 3. The landscape of prospective interventional trials is shown in Table 1. Some approaches showed promise in uncontrolled studies, but systematic evaluations of bortezomib [10], rituximab with or without intravenous immunoglobulin (IVIG) [11–13], clazakizumab [14], or imlifidase [15] have not led to breakthroughs, leaving insufficient evidence for clinical use. The large phase 3 clazakizumab trial (IMAGINE; ClinicalTrials.gov identifier, NCT03744910) was terminated due to lack of efficacy. Trials of complement inhibitors, such as C1 esterase inhibitors, the C1s antibody sutimlimab or antibodies targeting C5, including an early terminated eculizumab trial in C4d-positive AMR (NCT01895127), also lacked convincing efficacy [16–18]. However, case series suggested efficacy, particularly in early AMR [19]. Not all AMR cases are necessarily complement-driven, largely depending on the quantity of antibodies binding to the endothelium (i.e. antibody titers). Indeed, AMR can occur without complement activation, making complement inhibition only a partial solution at best. Splenectomy was previously proposed as a last-resort therapy for severe early AMR [20]. We believe its long-term risks, especially in young patients, warrant critical evaluation; therefore, we do not consider splenectomy in our recommendations.

**Table 1:** Systematic interventional trials in AMR.

Target	Treatment	Mode of action	Reported trial phase	Trial acronym	Identifier^a^	First author, year	Main trial results
DSA	Imlifidase	IgG cleavage	Phase 2 (finished)		NCT03897205	Halleck, 2024 [15]	Marked transient reduction in DSA levels. No effect on AMR morphology and graft survival
	Plasmapheresis	Ig depletion				Soullilou, 1983 [48]	No effect on graft survival
						Allen, 1993 [49]	No effect on graft survival
						Bonomini, 1985 [50]	Significant effect on graft survival
	Immunoadsorption	IgG depletion	Phase 2 (prematurely terminated)	AKARIS		Böhmig, 2007 [51]	Less graft failures in the treatment arm
	Efgartigimod	IgG degradation	Phase 2 (recruiting)	SHAMROCK	NCT06503731		Not yet finalized
Complement	BIVV009	Inhibition of C1s	Phase 1 (finalized)		NCT02502903	Eskandary, 2018 [18]	No effect on AMR activity
	BIVV020		Phase 2 (recruiting)		NCT05156710		Not yet finalized
	Eculizumab	C5 targeting	Phase 3 (finished)		NCT01327573	Kulkarni, 2017 [17]	No effect on biopsy results
			Phase 2 (prematurely terminated)		NCT01895127		Not published
	C1-INH	C targeting	Phase 2 (finished)		NCT01147302	Montgomery, 2016 [16]	Trend toward improvement in renal function. Less cg in 6-month biopsies
			Phase 3 (prematurely terminated)		NCT02547220		Not published
			Phase 3 (prematurely terminated)		NCT03221842		Not published
B cells	Rituximab	B cell depletion	Phase 3 (finished)	RITUX-ERAH	NCT01066689	Sautenet, 2016 [11]	No effect on graft loss or renal function
			Phase 2 (prematurely terminated)	TRITON	2010–023746-67	Moreso, 2018 [12]	No effect on biopsy results and eGFR
			Phase 4 (prematurely terminated)	RituxiCAN-C4	NCT00476164	Shiu, 2020 [13]	No effect on clinical outcomes
			Phase 3 (recruiting)	TAR:GET-1	NCT03994783		Not yet finalized
	Fostamatinib	SYK inhibition	Phase 2 (recruiting)	FOSTAMR	NCT03991780		Not yet finalized
Innate/adaptive immunity	IVIG plus steroids	Multiple targets	Phase 2 (finished)		ACTRN12612000252819		Not published
IL-6/IL-6R	Clazakizumab	IL-6 neutralization	Phase 2 (finished)		NCT03444103	Doberer, 2021 [14]	DSA reduction; modest effect on molecular AMR (12 mo); possible effect on eGFR slope
			Phase 2 (finished)			Jordan, 2022 [82]	Trend toward stabilization of eGFR; reductions in DSA/graft inflammation (uncontrolled trial)
			Phase 3 (prematurely terminated)	IMAGINE	NCT03744910		No effect on eGFR decline
	Tocilizumab	IL-6R blockade	Phase 3 (recruiting)	INTERCEPT	NCT04561986		Not yet finalized
PC	Bortezomib	Proteasome inhibition	Phase 2 (finished)	BORTEJECT	NCT01873157	Eskandary, 2018 [10]	No effect on DSA levels, morphologic/molecular biopsy results, and eGFR slope
			Phase 2 (finished)	TRIBUTE	NCT02201576		Finalized, but not yet published
			Phase 2 (recruiting)		NCT03737136		Not yet finalized
PC/NK	Felzartamab	CD38 binding	Phase 2 (finished)		NCT05021484	Mayer, 2024 [37]	Substantial effect on AMR activity; NK cell depletion; marked decrease in dd-cfDNA
	Felzartamab		Phase 3 (ongoing)	TRANSCEND	NCT06685757		Not yet finalized

cg, transplant glomerulopathy; C, complement; C1-INH, C1 esterase inhibitor; IL-6R, interleukin-6 receptor; PC, plasma cells; SYK, spleen tyrosine kinase.

^a^Provided are ClinicalTrials.gov, EudraCT or ANZCTR identifiers.

The lack of strong evidence has resulted in substantial heterogeneity in clinical practice. A 2023 online survey in Europe [21] indicated that over half of patients with chronic-active AMR receive no additional treatment beyond optimized immunosuppression. Common reasons highlighted in the survey to leave chronic-active AMR untreated, despite the known association with impaired graft outcome, include appreciation of disease irreversibility, fear of costs and side effects, and the lack of robust trial data. When additional treatments are used, IVIG, steroid pulses, and apheresis are common, whereas rituximab or other biologics are used less frequently [21].

#### Preventive measures

Preventing AMR is crucial in the absence of approved treatments. This begins with proper donor typing, optimal epitope matching, and identifying non-acceptable HLA antibodies to avoid harmful preformed DSA [22] and minimize immunologic risks, including *de novo* DSA formation [23]. Adequate immunosuppression is essential [24], with non-adherence remaining a major risk factor [6]. Regarded as the fifth vital sign in transplant recipients [25], adherence requires long-term physician support. Once-daily drug regimens [26] and “electronic” health solutions including mobile applications or video consultations [27] may improve self-management and medication adherence.

Regular *de novo* DSA screening may enable early intervention. A European Society of Organ Transplantation working group concluded that it could optimize long-term graft survival in patients without graft dysfunction, especially if an effective AMR treatment becomes available [28]. However, controversy remains. The OUTSMART trial randomized >2000 participants to receive serology-led care based on antibody results versus blinded standard care [29]. Such care, which included patient interviews to encourage medication adherence and optimization of tacrolimus-based immunosuppression, did not affect the rate of graft failure. While it reduced biopsy-proven rejection risk (HR: 0.50, *P* = .03), it had no effect on various other secondary outcomes, such as patient survival, levels of graft dysfunction, or medication adherence [29]. As discussed later in this article, in addition to the detection of DSA, other non-invasive monitoring tools—such as donor-derived cell-free DNA (dd-cfDNA) and urinary chemokines—are currently evaluated as non-invasive monitoring for the early detection of AMR [30, 31]. These will be discussed in more details next.

A detailed discussion of desensitization protocols for pre-sensitized patients, which, among others, may include antibody depletion using concepts such as the immunoglobulin G (IgG) cleaving enzyme imlifidase [32], is beyond this article's scope. However, we want to highlight a cohort study suggesting that prophylactic eculizumab can successfully prevent early AMR, although long-term follow-up and a randomized trial failed to show significant benefit [33, 34]. Based on a recent case report, a promising approach could involve anti-CD38 therapy in the context of desensitization for AMR prophylaxis through natural killer (NK) cell depletion, but long-term data are needed before recommending this strategy [35].

#### Expert consensus

A few years ago, expert consensus-based clinical practice guidelines for AMR treatment were published under the umbrella of The Transplantation Society (TTS) [3], summarized in Table 2. Evidence grades ranged from 2B to 3C (KDIGO codes), indicating a clear lack of strength in the experts’ recommendations. The guidelines emphasized that histology alone is insufficient to understand AMR pathophysiology. This is supported by recent evidence demonstrating that microvascular lesions, indistinguishable from those of AMR can be triggered independently of alloantibodies, which may have therapeutic implications [36] (see also our discussion of antibody-independent NK cell activation next). Additional clinical information (preformed vs. *de novo* DSA; early vs. late AMR) is therefore necessary. For early (<30 days) or late active AMR with preformed DSA (>30 days), plasmapheresis plus IVIG were recommended as standard of care (SOC). In severe cases with a high risk of graft loss or for concomitant TCMR, steroids were recommended. Suggested adjunctive treatments included complement inhibitors and splenectomy for early AMR cases, as well as and IVIG. In addition, optimizing baseline immunosuppression was recommended: adding steroids to steroid-free regimens and maintaining tacrolimus levels >5 ng/mL, as well as evaluation and management of non-adherence [3] (Table 2).

**Table 2:** TTS 2019 Consensus and the authors’ proposed treatment approaches, based on expert opinion (ungraded practice recommendations).

2019 Consensus	DSA	Banff 2017 phenotypes	SOC	Consider adjunctive therapies
Early acute (<30 days)	Preexisting DSA (or nonimmunologicaly naïve)	Active AMR	• Plasmapheresis/IVIG^a^ • Steroids	• Complement inhibitors • Rituximab • Splenectomy
Late (>30 days)	Preexisting DSA	Active AMR	• Plasmapheresis/IVIG^a^ • Steroids	• Rituximab
		Chronic AMR	• Optimize baseline immunosuppression^b^	
	*De novo* DSA	Active AMR	• Optimize baseline immunosuppression^b^ • Evaluate and manage non-adherence	• Plasmapheresis, IVIG • Rituximab
		Chronic AMR		• IVIG
**Authors’ proposal**	**DSA**	**Banff 2022 phenotypes**	**Primary treatment**	**Consider adjunctive therapies**
			**General considerations**:	
			Consider the following when deciding on the type and intensity of treatment: • Severity and acuity of graft dysfunction (e.g. activity index): May determine the intensity of the chosen treatment. • Level of chronic injury at the time of biopsy(chronicity index): this helps exclude unmodifiable chronic damage. • Presence of comorbidities: Avoids increased risks associated with over-immunosuppression. • Recipient age: Presumably, elderly patients derive less benefit. • If concomitant TCMR is diagnosed, also treatment with corticosteroids, ATG, or alemtuzumab should be considered. • Consider follow-up biopsies for early detection of phenotype transition (e.g. transition to active AMR after a diagnosis of probable or of probable AMR) or for treatment monitoring.	
Early (<6 months)	Preexisting (and/or *de novo*) DSA	Active AMR (chronic-active AMR)	• Apheresis (Plasmapheresis or immunoadsorption) with or without IVIG^c^ • Optimize baseline immunosuppression^b^	• CD38 antibody^d^ • Complement inhibition^e^
		Probable AMR	• Optimize baseline immunosuppression^b^	• CD38 antibody^d^
	No DSA	MVI, C4d-/DSA-negative	• Optimize baseline immunosuppression^b^	• CD38 antibody^d^
Late (>6 months)	*De novo* (and/or preexisting) DSA	Active/chronic-active AMR	• Optimize baseline immunosuppression^b^ • CD38 antibody^d^	
		Chronic AMR	• Optimize baseline immunosuppression^b^	
		Probable AMR	• Optimize baseline immunosuppression^b^	• CD38 antibody^d^
	No DSA	MVI, C4d-/DSA-negative	• Optimize baseline immunosuppression^b,f^	• CD38 antibody^d^

ATG, antithymocyte globulin; PP, plasmapheresis.

a Daily or alternate day plasmapheresis sessions × 6 based on DSA titer; IVIG 100 mg/kg after each plasmapheresis treatment or IVIG 2/kg at end of plasmapheresis treatments

b Includes the use of tacrolimus with goal through levels of >5 ng/ml and use of maintenance steroid equivalent to prednisone 5 mg/day.

c We suggest apheresis treatment courses over 2–3 weeks, starting with three daily treatments, followed by sessions every 2–3 days. Optionally, high-dose IVIG (2 g/kg) may be administered at the end of the apheresis course.

d CD38 targeting may include the use of daratumumab at 16 mg/kg; weekly over 1 month (pretreatment before the first two administrations to prevent first dose reactions), followed by monthly doses until months 3–6. The duration of treatment may be guided by dd-cfDNA monitoring and/or follow-up biopsy results.

e Complement inhibition may include the administration of one or more doses of eculizumab.

f In DSA- and C4d-negative MVI, a switch from mycophenolic acid to an mTOR inhibitor may be considered for optimization of maintenance immunosuppression.

#### Emergence of CD38-targeted treatment as a new option

Monoclonal antibodies targeting CD38, a surface molecule highly expressed by plasma cells and NK cells, have shown promise in AMR treatment, with positive results reported for daratumumab in case series and for felzartamab in a randomized phase 2 trial for late DSA-positive AMR [37, 38]. In the phase 2 trial, 82% of treated patients achieved morphologic AMR resolution by week 24, compared to 20% in the placebo arm, with a reduction in AMR-associated transcripts and dd-cfDNA. Felzartamab led to a substantial NK cell reduction, suggesting effector cell depletion, rather than plasma cell effects, mitigates DSA-mediated injury. In a recent re-analysis of gene expression patterns in trial biopsies, felzartamab was shown not only to selectively (and transiently) affect transcripts associated with AMR activity, but also—over the 12-month study period—to modulate molecular injury and repair responses [39]. Infusion-related reactions were common, but no severe infections occurred. In summary, the trial demonstrated the potential of CD38 targeting as a promising, safe AMR treatment, highlighting the role of NK cells in AMR, and supported dd-cfDNA as a non-invasive injury monitoring tool [37].

### Authors’ 2025 practice recommendations

**Figure 2:**
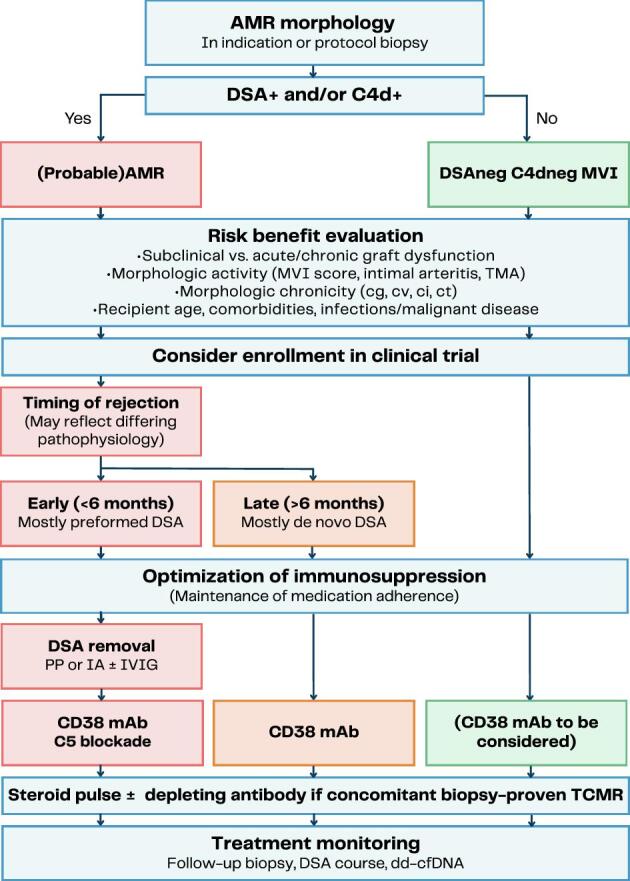
Proposed therapeutic algorithm for the management of AMR and DSA- and C4d-negative MVI. cg, transplant glomerulopathy; ci, interstitial fibrosis; ct, tubular atrophy; cv, vascular fibrous intimal thickening; g, glomerulitis; IA, immunoadsorption; PP, plasmapheresis; ptc, pertitubular capillaritis; TMA, thrombotic microangiopathy.

Acknowledging the lack of high-quality evidence, we propose a practical treatment algorithm for AMR, partly building on the 2019 expert consensus (Table 2, Fig. 2). Given the limited evidence supporting current therapeutic options, we encourage the inclusion of patients, when possible, in interventional trials evaluating innovative treatment concepts. We suggest that, as also considered in the 2019 consensus, the timing of AMR diagnosis, which is likely only a reflection of different contributions of various confounders (presence of ischemia/reperfusion injuries, anamnestic versus naïve humoral response, level of chronic damages … etc., see below), be considered a key determinant of the effectiveness of specific therapeutic strategies, such as DSA removal and complement inhibition. Early cases, which are often clinically overt and morphologically present as active AMR, may frequently be associated with preformed DSA acting on an activated graft endothelium due to ischemia/reperfusion. By contrast, later cases, which are often subclinical and present with chronic injury at diagnosis (e.g. chronic-active phenotype), may commonly arise from *de novo* DSA. The appearance of *de novo* DSA in circulation is likely more gradual, allowing time for the endothelium to adapt. [40]. To differentiate between early and late AMR and to account for potential differences in pathophysiologic, clinical, and morphologic presentation, we propose an arbitrary threshold of 6 months, which is primarily based on its use as a key eligibility criterion for “late” AMR in published randomized controlled interventional trials [10, 37, 38]. However, given the limitations of this approach, we acknowledge that this is not a strict recommendation and, as a reference point, can be applied flexibly in specific cases. For example, it should be noted that early rejection may result from *de novo* DSA, whereas late rejection can occur due to persistence of preformed DSA. The major components of our practice recommendations include a careful risk–benefit evaluation, followed by consideration of causal therapy targeting DSA and treatments aimed at downstream immune activation (Table 2, Fig. 2).

### Estimating risk–benefit to guide treatment intensity

Before selecting treatment options, we recommend evaluating the course of kidney function and proteinuria besides disease activity, chronicity, and recipient factors to guide decisions on the type and intensity of treatment. This also applies to concomitant TCMR, where pulse steroids or depleting antibodies may increase the risk of over-immunosuppression (Table 2, Fig. 2).

#### Disease activity

We suggest adjusting treatment based on the extent and kinetics of graft dysfunction and morphologic disease activity. Subclinical and lowly inflamed AMR, e.g. diagnosed in a protocol biopsy, requires likely less aggressive therapy than a high-grade inflammation, or even the presence of intimal arteritis or thrombotic microangiopathy, in indication biopsies. In this respect, the use of activity indices, which, independently of the rejection (sub)category, may mirror the intensity and clinical impact of rejection processes, could become of help. Their validation and assessment of clinical value is the primary objective of a working group recently launched at the Banff 2022 meeting [41].

#### Chronic injury

We also suggest evaluating the level of chronic injury on biopsy, a major predictor of shortened graft survival reflecting the renal function reserve at baseline [42]. This assessment—along with proteinuria levels, which indicate the extent of chronic glomerular injury—is crucial for avoiding unnecessary treatment in cases of likely irreversible severe damage. However, the possibility of sampling error must be considered when interpreting biopsy results. Similar to the activity index, the chronicity index proposed by the Banff Working Group may aid clinical decision-making and help assess treatment futility [41].

Currently, few validated biomarkers beyond biopsy findings are available to reliably predict allograft outcomes in clinical practice. One promising tool, the iBox prediction score, has been developed for estimating allograft survival and incorporates various parameters including the level of DSA as non-invasive marker [43]. However, the utility of such tools in guiding clinical decision-making—particularly in the context of AMR—remains to be established.

#### Recipient age and comorbidities

In a risk–benefit analysis, we suggest considering recipient factors—particularly age and comorbidities, including infections or malignant disease—that increase the risk of complications from intensified immunosuppression. In large transplant populations, recipient (and donor) age was shown to significantly raise the risk of death with a functioning graft [44]. As a consequence, elderly patients—where AMR may not be the primary contributor to graft failure—who receive an old graft (with donor-inherited chronic damage) may derive less benefit from intensive treatment while still being exposed to the risks associated with intensified immunosuppression [45].

### Causal therapy

The approach of treatments aimed at addressing the underlying cause is often preferred; in AMR, this would be targeting the antibodies (Table 2, Fig. 2).

#### Antibody removal

We recommend DSA removal as a mainstay treatment for early AMR, regardless of whether the DSA is preformed or *de novo* (although most cases are linked to preexisting DSA, and even cases without detectable preformed DSA may involve donor-specific B cell memory). Antibody removal can be achieved through plasmapheresis, as per the 2019 TTS expert consensus, or through immunoadsorption. The recommendation for plasmapheresis is based on findings from uncontrolled series [46, 47], although early controlled trials—conducted before clear-cut AMR criteria were established—have yielded conflicting results [48–50]. Support for immunoadsorption comes from a small trial evaluating protein A-based antibody depletion in severe C4d-positive AMR, which demonstrated a benefit in rejection reversal [51]. However, the authors of this article are aware that the availability of immunoadsorption may be limited and its use not widespread. Moreover, no head-to-head trial comparing different apheresis techniques is currently available. Apheresis sessions may be scheduled daily for the first 3 days, then every 2–3 days over 2–3 weeks. The additional use of IVIG can be considered, but it has weak supporting evidence, although a retrospective study suggested potential benefit [52]. For pathophysiologic considerations, we recommend using high-dose IVIG at the end of the apheresis course (e.g. 2 g/kg), rather than low doses after each treatment session. The concept of (transient) antibody depletion may have limitations, as demonstrated by a recent trial comparing the IgG-cleaving enzyme imlifidase with plasmapheresis plus IVIG [15]. In this trial, imlifidase—established as a therapeutic option for desensitization of highly sensitized transplant recipients [32]—did not demonstrate an advantage over plasmapheresis in the treatment of AMR, despite a demonstrated profound impact on DSA levels. Notably, despite adjunctive treatment with steroids and rituximab, neither approach led to a meaningful reduction in MVI on follow-up biopsies. Without an untreated control group, however, the true impact on rejection activity and progression remains unclear [15].

#### Optimized immunosuppression and maintenance of medication adherence

For all AMR phenotypes, we recommend optimized immunosuppression (along with maintenance of medication adherence), in line with the 2019 TTS expert consensus. This recommendation is supported by data showing that reduced immunosuppression (whether from non-adherence or physician-directed changes) increases the risk of DSA formation and AMR occurrence [53]. In this context, it is important to note that maintenance immunosuppression is typically adjusted based on drug trough levels (a pharmacokinetic approach), whereas an ideal strategy would incorporate a functional assessment of immune suppression (a pharmacodynamic approach). An interesting strategy in this context may be monitoring of Torque Teno virus (TTV), a ubiquitous, non-pathogenic virus whose replication is controlled by the host immune system. Elevated levels of TTV DNA in plasma have been associated with increased levels of immunosuppression. As such, TTV DNAemia is being investigated as a dynamic and quantifiable biomarker of net immune function, particularly in transplant recipients [54, 55]. However, standardized thresholds and clinical interpretation remain to be fully validated in larger, prospective studies.

### Targeting downstream immune activation.

While causal therapy targeting the antibodies seems a rational approach to the treatment of AMR, evidence of clinical efficacy of such causal therapy is very limited. Alternative approaches to treating AMR could be considered, targeting the downstream antibody-dependent cellular cytotoxicity and antibody-mediated complement activation (Table 2, Fig. 2).

#### CD38 targeting antibodies

We suggest CD38 antibody therapy (e.g. off-label use of daratumumab, depending on availability) in addition to apheresis in early AMR and recommend it as sole therapy alongside optimized immunosuppression in late AMR, preferentially in cases with limited chronicity score. CD38-targeting effects may not be durable in some recipients, indicating that prolonged treatment courses of six or more months may be necessary [38]. The optimal treatment schedule and long-term side effects remain unknown and should be considered when using a CD38 antibody. Additionally, the optimal strategy for guiding treatment—potentially involving biomarkers—is still under evaluation.

#### Complement inhibition

Based on observational results [20], we suggest the use of eculizumab in early severe (refractory) cases of AMR, which may include cases associated with thrombotic microangiopathy.

### Treatment monitoring

For guiding treatment, we suggest using monitoring strategies beyond regular kidney function assessments, such as DSA including their complement-fixing capability [56], and, if available, dd-cfDNA evaluation, along with follow-up biopsies [57] (Table 2, Fig. 2). While evidence supporting specific AMR treatment options is limited, the optimal duration remains even more uncertain. For example, the TTS guidelines suggested four to six sessions of plasmapheresis for active AMR, but without supporting evidence. Antibody rebound can be expected after cessation of plasmapheresis. Similarly, the felzartamab trial suggests CD38 targeting effects may not be durable [38]. Monitoring dd-cfDNA, either as a relative fraction or, to account for variations in total cfDNA, as an absolute concentration, may be a useful non-invasive tool for assessing ongoing allograft injury, as it shows a strong correlation with AMR-associated gene expression and rejection morphology [30, 58, 59]. In a recent trial of 40 DSA-positive patients comparing dd-cfDNA-guided versus clinician-guided biopsy management, dd-cfDNA detection enabled significantly earlier AMR diagnosis (median 2.8 vs. 14.5 months) [60]. Additionally, longitudinal monitoring demonstrated a 77% positive predictive value and an 85% negative predictive value for AMR [60]. Finally, there may be a potential of dd-cfDNA as a monitoring tool to detect the treatment effects of CD38 targeting [37]. However, further research is necessary to clarify the clinical value and cost-effectiveness of dd-cfDNA monitoring.

A detailed discussion of non-invasive biomarker assessment in general—including its various clinical applications, such as early rejection diagnosis, outcome prediction, and treatment monitoring—may be beyond the scope of this article. Potential candidates for improving diagnostic precision could include, for example, the measurement of C-X-C motif chemokine 10 in urine. However, despite its promise as a future strategy, the clinical value of chemokine monitoring remains controversial and requires further validation [31, 61]. Reflecting current uncertainties, the recently published multicenter EU-TRAIN study found that a range of biomarkers—including blood messenger RNAs and non-HLA antibodies—failed to improve rejection prediction beyond SOC monitoring parameters and anti-HLA DSAs [62].

## NEW DEVELOPMENTS

### Diagnosis

The Banff classification is regularly updated, following evolving insights in the pathobiology and phenotypic spectrum of rejection. In the Banff 2022 scheme, two separate subcategories have been introduced in the overarching AMR/MVI category: “MVI, DSA-negative, C4d-negative” and “probable AMR,” the latter characterized by subthreshold MVI in patients with circulating DSA [4]. The DSA-/C4d-negative phenotype shares molecular similarities with AMR [63, 64] but may be pathophysiologically distinct [65]. A re-analysis of such cases suggests some involve DSA binding, while others arise from DSA-independent mechanisms [66]. A study of >16 000 renal allograft biopsies across 30 centers found AMR had a hazard ratio of 2.7 for graft failure, while DSA-negative MVI had a hazard ratio of 2.1 [67]. MVI cases that were DSA- and C4d-negative had an increased risk of developing full AMR and transplant glomerulopathy. In this study, probable AMR was not significantly linked to graft failure (hazard ratio 1.7) but had an intermediate risk between no rejection and full ABMR [67]. In recent years, numerous studies have demonstrated added value of biopsy-based transcriptome analysis in AMR, including the Molecular Microscope Diagnostic platform [68] and the BHOT platform developed by a Banff Working Group [63]. While validated molecular analysis is included in the Banff scheme, it is not yet widely available [69]. Transcriptomics may provide prognostic insights, as shown in late AMR cases, where injury/repair-associated transcript sets correlated with accelerated estimated glomerular filtration rate (eGFR) decline [70].

### Pathophysiology

The conventional view of DSA-triggered inflammation includes complement activation, endothelial effects via HLA signaling, and Fc receptor-dependent mechanisms [1]. Recently, NK cells have emerged as key effectors of injury, triggered by endothelium-bound DSA and Fc gamma receptor IIIA [1]. This is supported by the predominance of NK cell transcripts [71, 72] and morphologic NK cell accumulation [73], as well as associations between NK cell genetics and MVI [74]. NK cells may also contribute to rejection phenotypes beyond classical AMR, including the DSA-negative, C4d-negative MVI phenotype [63, 65, 75]. DSA-independent NK cell activation may involve activating receptors or HLA class I mismatches in association with inhibitory KIR receptor patterns [65, 76]. The suggestion that NK cells play a role in all causes of MVI, also in the absence of DSA, has important therapeutic implications: specifically targeting these cells—such as with CD38 antibodies [37, 38]—not targeting the initial disease cause, but the downstream, less-specific immune activation.

### DSA-negative and C4d-negative MVI

Managing non-AMR phenotypes presents additional challenges, particularly in cases such as C4d or DSA positivity without MVI, probable AMR with subthreshold MVI, or MVI with both C4d and DSA negativity. In the latter scenario, a thorough re-evaluation of immunologic data is warranted to definitively exclude AMR, including assessing for low-level DSA, shared eplets, or potential non-HLA reactivity. For both MVI sub-phenotypes, close clinical and serologic monitoring is recommended, with follow-up biopsy considered to detect potential progression to AMR. If probable AMR is confirmed, treatment should align with established AMR protocols. Conversely, in cases of MVI without C4d and detectable DSA, therapies targeting DSA should be avoided. However, optimizing maintenance immunosuppression through dual inhibition of calcineurin and mTOR has shown promising effect in both reducing MVI and preventing graft loss in a pilot clinical study [65]. Given that these pathological conditions converge on a common final pathway involving NK cell activation and subsequent MVI—and that depleting NK has been proven efficient in murine experimental model of missing self-induced, NK cell-mediated rejection [65]—exploring anti-CD38 therapy appears to be a rational approach (Table 2, Fig. 2).

### New treatments under investigation

#### Felzartamab

Based on promising results phase 2 trial results, the CD38 antibody felzartamab received orphan drug designation for AMR treatment from the US FDA and the European Commission [37, 38]. In addition, the FDA granted it breakthrough therapy designation. An extension of the phase 2 trial is currently exploring personalized treatment regimens using dd-cfDNA monitoring, and a phase 3 trial has been initiated (TRANSCEND; NCT06685757), aiming for the first approved AMR treatment [38].

#### Tocilizumab

Interest in the anti-IL-6 receptor antibody tocilizumab grew after a study showed it stabilized renal function, modulated DSA levels, and improved histology in 36 kidney transplant patients with chronic AMR [77]. Despite setbacks from the IMAGINE clazakizumab trial, a phase 3 multicenter trial is currently investigating tocilizumab in chronic-active AMR [78].

#### High-dose IVIG

The role of high-dose IVIG in AMR has not yet been systematically studied. The efficacy of treatment with IVIG over a period of up to 6 months, together with steroid pulse therapy, was addressed in a randomized open-label trial conducted in Australia. Trial results have not yet been published (ANZCTR identifier, ACTRN12612000252819).

#### Efgartigimod

By blocking neonatal FcR receptor-mediated IgG recycling, efgartigimod, an FcRn antagonist, approved for myasthenia gravis [79], may reduce IgG and potentially mitigate AMR. A phase 2 trial in late AMR is underway (SHAMROCK; NCT06503731).

#### Fostamatinib

Experimental data from sensitized rats have suggested that the spleen tyrosine inhibitor fostamatinib is able to prevent DSA production [80]. A phase 2 trial is currently evaluating its use in chronic-active AMR (NCT03991780).

#### BIVV020

A phase 2 trial is currently underway, assessing BIVV020, a next-generation anti-C1s antibody blocking the classical complement pathway, in preventing and treating AMR on top of SOC treatment (NCT05156710).

### Future trial design

Designing interventional trials for AMR is challenging and requires a robust study design to reliably assess the safety and efficacy of new treatments.

#### Target population

Overly strict eligibility criteria can reduce representativeness, complicate recruitment, and risk trial delays or premature termination. Another challenge is the evolving AMR definitions in updated Banff schemes, which can affect phenotypic categorizations, allograft survival predictions, and result interpretation. Specifically, the subdivision of AMR into active, chronic-active, and chronic subcategories does not fully reflect the actual disease stage. Activity and chronicity indices within the broader AMR category could be more informative and guide eligibility criteria [41]. The scientific exchange with the European Medicines Agency led to a more restricted AMR definition, which may be better suited for clinical trials [81].

#### Control arm

Since no treatment for late AMR is approved, a placebo arm with optimized baseline immunosuppression may be ethically justifiable in smoldering late AMR cases. However, for clinically overt early AMR with high graft loss risk, a placebo design may be inadequate, and SOC therapy (e.g. apheresis) may need to be included, with the investigational drug tested against a placebo as an add-on.

#### Endpoints

Assessing hard endpoints such as graft survival requires large, long-term, multicenter trials. The IMAGINE trial (NCT03744910), designed to evaluate clazakizumab in chronic-active AMR with graft survival as the primary endpoint, aimed to enroll 350 patients. To reduce sample size and trial duration, eGFR slope was chosen as a surrogate endpoint for potential early approval. However, an interim analysis of 200 patients showed no meaningful differences, leading to trial termination. A useful strategy is designing smaller phase 2 trials to optimize safety and surrogate efficacy data, including MVI evolution, molecular signatures, and non-invasive biomarkers. If promising, results can inform phase 3 trial designs. An example is the felzartamab phase 2 trial, which led to the larger phase 3 TRANSCEND trial (NCT06685757). The FDA's approval of histologic AMR activity as a 6-month endpoint for this trial may help reduce sample size and shorten trial duration in similar studies.

## SUMMARY

AMR remains a major challenge, linked to poor graft survival despite various treatments, none of which are approved for clinical use. In this expert opinion paper, we recommend optimizing baseline immunosuppression across different MVI phenotypes. Apheresis or complement inhibitors may be reserved for early AMR, steroid pulses for concomitant TCMR. While several trials evaluating different therapeutic concepts have yielded disappointing results, anti-CD38 therapy has emerged as a promising new strategy. By depleting NK cells, this strategy can disrupt AMR activity, as suggested by a recent exploratory phase 2 trial. The results of this randomized trial, together with encouraging results from anecdotal reports and case series, have led the authors to propose CD38 targeting as an adjunctive treatment across different AMR phenotypes, and maybe even in case of MVI without C4d nor DSA. However, long-term safety data in transplantation are lacking, and the results of an ongoing phase 3 trial are awaited. Several novel strategies are currently under investigation, offering hope for better management.

## Data Availability

No new data were generated or analyzed in support of this research.
